# The social and health protection of migrants in Chile: qualitative analysis of civil society proposals for constitutional change

**DOI:** 10.1186/s12889-023-16093-w

**Published:** 2023-06-22

**Authors:** Sophie Esnouf, Alice Blukacz, Alexandra Obach, Edward Mezones-Holguin, Manuel Espinoza, Jocelyn DeJong, Baltica Cabieses

**Affiliations:** 1 Centre of Global Intercultural Health (CeSGI), Universidad del Desarrollo, Santiago, Chile; 2 Centro de Excelencia en Investigaciones Económicas Y Sociales en Salud, Universidad San Ignacio de Loyola, Lima, Perú; 3 Epi-Gnosis Solutions, Piura, Perú; 4 ETESA UC, Departamento de Salud Pública, Facultad de Medicina, Pontificia Universidad Católica de Chile, Santiago, Chile; 5 Department of Epidemiology and Population Health at the Faculty of Health Sciences, American University of Beirut, Beirut, Lebanon; 6 Department of Health Sciences, University of York, York, England

**Keywords:** Migrants, Migration, Public health, Civil participation, Democracy, Latin America, Constitution, Reform, Social protection

## Abstract

**Background:**

A sustained period of social, economic, and political unrest took place during October of 2019 in Chile. As an institutional solution, the “Agreement for Social Peace and the New Constitution” was signed. In this document, most political parties committed to reestablishing peace and public order in Chile, agreeing on the initiation of a constitutional process. To promote participation of civil society actors, the “Popular Initiative for Norms” was enabled. This was a platform where civilians could submit proposals for constitutional norms to be discussed by the Constitutional Convention. We aimed to analyze proposals related to migrants and migrant health.

**Methods:**

We conducted a qualitative thematic analysis of the proposals. Sixteen of them were related to migrants, and we analyzed their association to health. We also evaluated their link to the Health Goals 2030 set out by the Chilean Ministry of Health and the Global Action Plan 2019–2023 for Promoting the Health of Refugees and Migrants by the World Health Organization.

**Results:**

Four main thematic categories were identified: 1) Humans rights of migrants, refugees, and asylum seekers; 2) Nationality and regularization of migrants and refugees; 3) Political participation and cultural integration of migrants and refugees; and 4) Specific regulations on slavery and human trafficking. These resonated with broader frameworks established in the Health Goals 2030 (Chile) and the Global Action Plan 2019–2023 for Promoting the Health of Refugees and Migrants by the World Health Organization.

**Conclusions:**

The ‘Popular Initiative for Norms’ was a non-binding participatory mechanism. Although the proposals sent through were not guaranteed to be included in the constitutional draft—and despite the final draft being rejected last September 2022—the platform allowed to gain insights into civilian opinions. Our findings showed that there is an incipient yet weak recognition of the rights and situation of migrants in Chile. There was no direct mention of health nor an explicit contemplation of social determinants of health. Despite there being an urgent need to define strategies for migrants’ health in Chile, this study demonstrated that civil awareness and interest are still insufficient.

**Supplementary Information:**

The online version contains supplementary material available at 10.1186/s12889-023-16093-w.

## Background

Worldwide, a total of 214 million people is estimated to be on the move today [1]. Although human mobility is not a new phenomenon, the magnitude and scale of migratory flows that we are currently witnessing is unprecedented [1]. One of the dimensions that has been given increasing attention with regards to migration is health [2], and the IOM has described migration as a key determinant of health and well-being. Migration can represent an important life event during which inequities linked to other social determinants of health such as gender, socioeconomic circumstances, education, or ethnicity may be exacerbated, or on the contrary, improved, depending on the reasons for migrating, the conditions of transit, and the context of the country of arrival and settlement [3]. In the different stages of migration, individuals can undergo physical and emotional stress, conveying a higher risk of contracting communicable diseases, accidental injuries, unwanted pregnancy, or delivery-related complications, and various noncommunicable diseases, as well as adverse mental health outcomes. Other factors associated to worse health outcomes for refugees and migrants are discrimination, poor living conditions, and barriers to accessing health services [3]. These are related to the social position of migrants which, is in turn, shaped by immigration status or precarious migration trajectories [4, 5]. Some of these adverse events and outcomes can be mitigated by social and health protection. Social protection is defined by the World Bank as “systems, policies, and programs that help individuals and societies manage risk and volatility and protect them from poverty and destitution—through instruments that improve resilience, equity, and opportunity” [6]. From a social determinant of health perspective, social protection has a positive impact on health, however, there are many barriers for migrants to access it, including barriers linked to migratory status, lack of information, and lack of schemes responding to their specific needs [7].

### Migration in Chile and Latin America

In Latin America, economic disparities and lack of work opportunities have fueled a rise in migration fluxes within the region during the last two decades, with the main destination being the ‘South Cone’: Argentina, Chile, and Brazil. One of the main countries of origin of migrants in the region is Venezuela, where the economic crisis, shortage of food and medical attention, and political polarization have led to 4.1 million Venezuelan refugees and migrants being forced to live outside their country as of July 2021, with 2.8 million in Colombia, 1.29 million in Perú and 455,000 in Chile [8].

According to data from the International Organization for Migration (IOM), between 2018 and the first half of 2022, Chile has become one of the countries with the largest migratory growth in the world [9]. In 2019, approximately 8% of the resident population was foreign, mainly from Peru, Venezuela, Colombia and Haiti [10]. This trend has only increased since 2020, due both to the COVID-19 pandemic and to the escalation of economic and political crises in neighboring countries [11].

Temporary visas can be granted for family reunification, employment, and investment, among others. Most visa applications fall under these categories, however the number of asylum applications has grown since 2015, when 623 applications were submitted. In 2018, 5727 were registered, however numbers fell again in 2019, and 1628 applications were reported for 2020. It is important to note that of the 19339 asylum claims registered between January 2010 and June 2021, only 689 have been accepted. Finally, in the first semester of 2021, the main country of origin for asylum applicants in Chile was Venezuela (73%) and among the 7 people whose claim was accepted in 2020, 4 were Venezuelan nationals [12]. In terms of international frameworks, Chile is signatory of the Geneva Convention of 1951 and of the Cartagena Convention on Refugees of 1984, however it is not signatory of the Global Compact for Migration of 2018.

Although international migrants, refugees and asylum seekers are a diverse group in terms of migratory status, country of origin, and socioeconomic level, foreign-born people in Chile tend to experience more multidimensional poverty than their local counterpart, 24% compared to 20% at national level, despite higher educational levels,and a higher proportion of this population group being included in the labour market than the Chilean population [13]. Multidimensional poverty explores 5 dimensions: education; health; work and social security; housing and infrastructure; and networks and social cohesion [14].

Considering this context, and that migration is a social determinant of health, it becomes necessary to establish public policies that specifically address this group of people, with a focus on social determinants of health and public health promotion, where efforts are tailored towards ensuring equitable access to quality health services, free of discrimination, exclusion, and stigma [15]. In Chile, although significant efforts have been made by successive governments to lower the administrative barriers to access healthcare faced by international migrants, they still face barriers linked to discrimination, a lack of adequate information on the right to health regardless of migratory status, and a lack of culturally relevant care.

### Chile’s current sociopolitical context

Triggered by a 30 peso (about USD 0.20) rise in the cost of public transport, a sustained period of social, economic and political unrest took place during October of 2019 in Chile, known as the “Social Outburst'' or *Estallido Social*. Mobilization and strikes revolved mainly around deep inequalities concerning pensions, health and education [16]. However, these demonstrations quickly escalated into public disturbances, violence, repression, and serious human rights violations, bringing tension around security matters and (mis)trust in political authorities. In response, the majority of Chile's political parties came together to find a democratic and institutional route out of the crisis. The result was the formulation of the “Agreement for Social Peace and the New Constitution'' (*Acuerdo Por la Paz Social y la Nueva Constitución*) [17], signed on the 15th of November 2019. In the Agreement, parties committed to reestablishing peace and public order in Chile and agreed on the initiation of a constitutional process, which could potentially culminate in replacing the present-day constitution written in 1980, under the dictatorship of Augusto Pinochet.

Citizen participation has had a central role in Chile’s constitutional process, shaping its evolution: from the demonstrations that questioned constitutional change in 2019; to the referendum that enabled its initiation; and finally, to the election of the body in charge of writing the draft. During the drafting period, different mechanisms were put in place to promote the continuity of citizen participation. One of these was the Popular Initiative for Norms, a virtual, online space where individuals or civil society groups could submit contributions and ideas to be discussed by the Convention, bringing forward issues meaningful to the public. Physical centers were enabled so that those with no internet access could inscribe their proposals manually. To be eligible to participate, people were required to be above 16 years of age, of Chilean nationality, foreign nationality with Chilean residence, or Chileans living abroad. They also had to register in Single Register of Popular Participation (*Registro Único de Participación Popular*) [18], which could be done online or at one of the enabled centers.

A total of 6,114 proposals were sent through the platform and underwent an admissibility revision, after which 2,496 were approved and published for the general public to vote. The admissibility revision was presided by the Popular Participation Commission (*Comisión de Participación Popular*), where they excluded proposals that went against statal obligations assumed by the Human Rights International Treaties and proposals that were impertinent to the constitutional debate, as well as those that were incomplete in its form.

The data was available on an online website, where it was possible to visualize all the proposals and the number of votes held by each. Following the two-week long voting period—where each person could vote for a total of 7 initiatives—77 proposals (3%) reached the required 15,000 votes or more to pass on to being discussed. Of these, 60 were supported by organizations or institutions and 17 by individuals [19].

### Civil society proposals related to migration and health

The proposed Constitutional document was finally rejected by the population that voted in the September 2022 referendum, however, we consider that the democratic process of soliciting proposals from civil society, especially given wide participation, provides insights into the population’s interests, needs and vision concerning migrants and their integration into the Chilean society.

Given that migration from Latin America and the Caribbean is a relatively new phenomenon in Chile, but of growing and significant magnitude, the findings of this study can be useful to get a sense of how migrants and migratory issues are currently perceived by civilians and organizations in times of political upheaval, and to analyze if health concerns are taken into consideration. We therefore decided to focus our analysis on proposals associated with migrants and refugees with a public health perspective. Additionally, we linked these proposals to both national and international guidelines, to explore the extent of alignment.

## Methodology

### Study design

We conducted a qualitative thematic analysis of migrant-related civil proposals uploaded to the official website for Popular Participation during the constitutional process in Chile.

### Data source

Data was extracted from the “Digital Platform for Popular Participation” a free access website, created to enable civil society participation during the Chilean constitutional process. Individuals and civil society organizations could send a maximum of 7 proposals for constitutional norms. To be eligible to participate, individuals or group representatives had to be over 16 years of age and of Chilean nationality, or foreigners with residency in Chile, or Chileans living abroad. Additionally, they required having a national identification card or to be registered with “clave única”, a digital password that allows access to state procedures [18]. The approved proposals were published in the section “Popular Initiative for Norms”. Each proposal was uploaded into one of the following categories, with the respective number of proposals in each one as follows: 1) Political system (452); 2) Constitutional principles (191); 3) State form (272); 4) Fundamental rights (998); 5) Environment (299); Justice system (146); and 6) Knowledge/Education systems (138).

### Search strategy

The keywords ‘migration’, ‘migrants’, ‘nationality’ and ‘multicultural’ were used in the website's search engine, which displayed the proposals containing these words in any part of the text. The data search and the data retrieval were conducted by SE and BC. The data search and the selection were conducted between January and April 2022.

### Selection criteria

A total of 31 proposals were obtained. A first filter was applied based on the title of each proposal, excluding those duplicated and those not relevant to the subject, after which 16 remained. Subsequently a complete reading of these proposals was made, and all 16 proposals were fully analyzed for extraction purposes.

### Extraction and synthesis of data

Data was extracted using Microsoft Excel, and classified by i) general characteristics of the proposal (Proposal number, amount of votes, author/name, supporting institution, three key words, central ideal of the proposal, audience it would benefit); ii) association with public health (does in mention public health in the title? yes/no, Does it mention public health within the text? yes/no; iii) Mention of Essential Public Health Functions (EPHF) as established by the WHO/PAHO, 2020; and iv) Mention of additional elements: “intercultural”, “gender”, “right”, “vulnerability” or similar, “global”, “social security”, “health system”, “social” or “civil participation”. This table is available in Spanish, in Supplementary File 1.

### Data synthesis and display

**Fig. 1:**
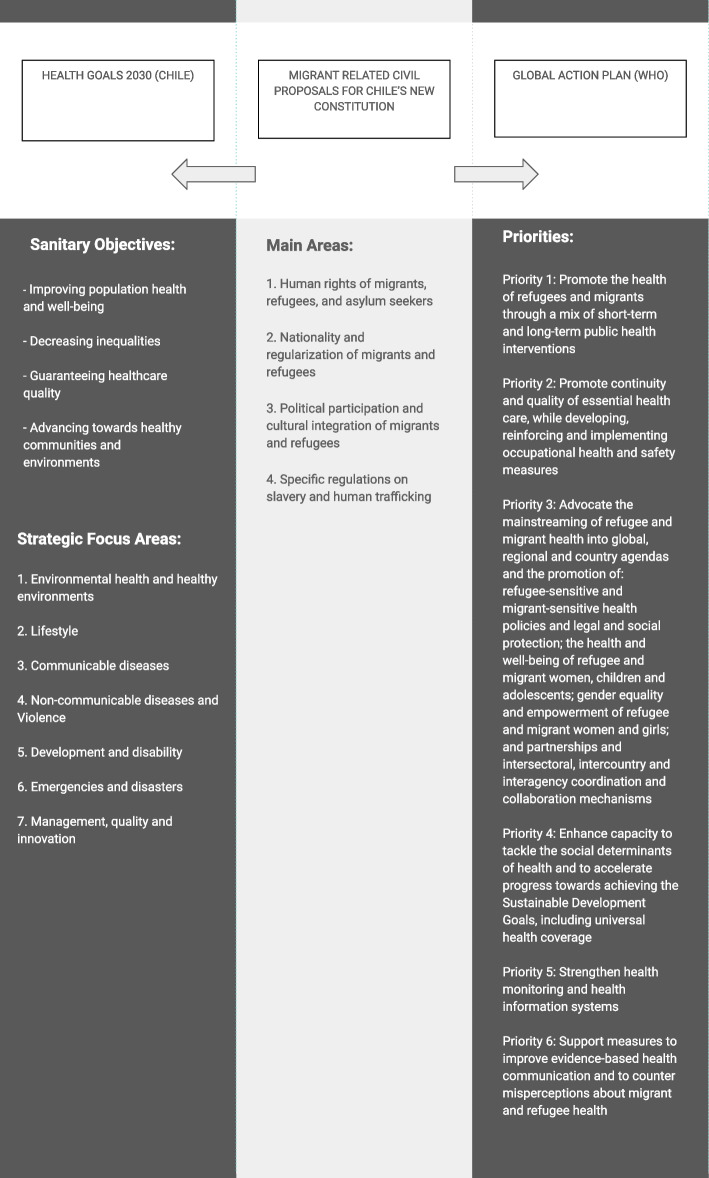
Migrant related civil proposals for Chile’s new constitution, Health Goals 2030 and its Strategic Focus Areas, and WHO Global Action Plan Priorities 2019 – 2023

Based on the dominant themes inherent in the data, four thematic categories were reached by SE, using an inductive approach [20], and revised by BC. These categories were: 1) humans rights of migrants, refugees, and asylum seekers; 2) nationality and regularization of migrants; 3) political participation and cultural integration of migrants and refugees; and 4) specific regulations on slavery and human trafficking. Each proposal was then linked to i) the corresponding Strategic Focus Areas of the Health Goals 2030, established by the Chilean Ministry of Health (MINSAL) [21] and ii) the priorities set out in draft Global Action Plan 2019–2023 for Promoting the Health of Refugees and Migrants by the World Health Organization (WHO) [15]. The detailed information is presented alongside the full list of proposals in Supplementary file 1. Figure 1 illustrates the alignment of the proposals with the Health Goals 2030 and its Strategic Focus Areas as well as the WHO Global Action Plan Priorities 2019 – 2023.

## Results

**Fig. 2:**
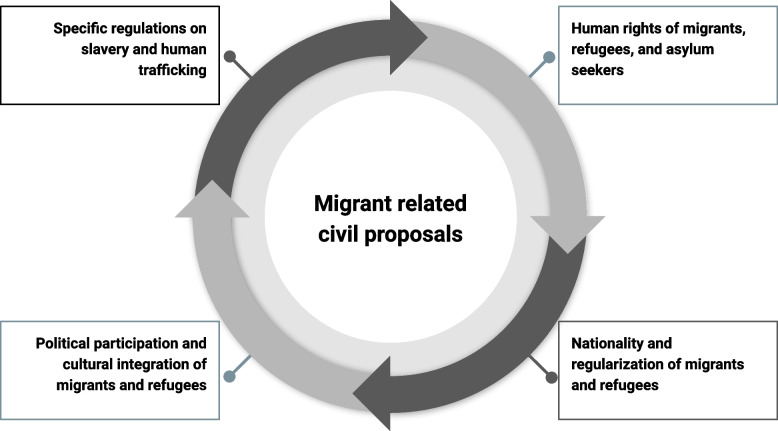
Main categories of migrant-related civil proposals for the new Constitution

The main themes identified were: 1) Humans rights of migrants, refugees, and asylum seekers; 2) Nationality and regularization of migrants and refugees; 3) Political participation and cultural integration of migrants and refugees; and 4) Specific regulations on slavery and human trafficking (Fig. 2).

Of the proposals we analyzed, 6 were supported by civil organizations, all of which are non-governmental organizations (NGO). Of these, the ‘Jesuit Migrant Service’ (Servicio Jesuita Migrante (SJM)), holds the longest trajectory, dating its origins to 1980, when it began as a volunteer-run religious group that lent general assistance and guidance to newcomers. It posteriorly professionalized and now holds a strong presence in the media, carrying out investigative work, advocacy, and judicial consulting. The ‘National Board of Migrants and Refugees’, ‘Chile Migrates’, and ‘Migrates Chile’ are all recent organizations (founded 2020–2021) that advocate for migrant and refugee human rights and help individuals in administrative and legal matters. ‘Liberate’ is a women led NGO born in 2015 that advocates, educates, and investigates on human trade and trafficking, as well as legally and psychologically aiding victims. Lastly, a conglomerate of feminist, gender activist NGOs from the Bio-Bio region (south of Chile) united to support one proposal.

### Category 1: humans rights of migrants, refugees, and asylum seekers

Seven proposals were grouped into this category. They all refer to the protection of the human rights of vulnerable groups within society, some from a broad perspective and others referring particularly to migrants, refugees, and asylum seekers, with a shared stance of being supportive of migrants.

From a broad perspective, the proposal “No to racism” (1.1) (55 votes) aims to “*ensure the right to equality*” by “*establishing a country with no prejudice and discrimination and including it in the constitution”*, which in turn *“will allow to formulate laws that penalize racism, building a better society*”. This proposal has a particular focus on physical appearance, highlighting the presence of discrimination that exists towards “*dark skinned”* migrants in Chile, in comparison to *“blonde”* foreigners. It also touches on the discrimination present towards darker skinned Chileans and indigenous groups. Amongst the proposals associated to migration, this was the only one that alluded to racial issues.

“Guarantee of the rights of people in a situation of structural vulnerability” (1.2) (135 votes) affirms that the State must recognize the existence of a structural violation of human rights affecting vulnerable minorities, including women, sexual dissidences, migrants, amongst others. It suggests that the Constitution must *“establish a normative and political framework that ensures the respect and guarantee of these peoples’ rights in an order of non-discrimination”* and must confront the structures that perpetuate this situation.

The initiative “Principle of intersectionality in the judicial system” (1.3) (1,234 votes) follows a similar line and focuses on unequal access to justice. Although it is mainly centered on women and mothers, it includes all minority groups that have historically been discriminated against. It brings forward the idea that, to protect human rights, the judicial system must incorporate the concept of intersectionality, meaning to “*respect all people according to their own realities (…) with a focus on gender and equity”*.

Three proposals refer particularly to the rights of migrants and asylum seekers. “Promotion and defense of the rights of migrants, asylum seekers and refugees” (1.4) (127 votes) suggests that integration and inclusion should be reached by the development of appropriate public policies, whereas two others refer particularly to the right to migrate and receive asylum.

One of these proposals, “Constitutional recognition of migration as a human right and the rights of migrants and refugees in Chile and of Chileans living abroad” (1.5) (5,794 votes) states that *“the new constitution must recognize the right of every person to migrate from and to Chile, respecting Human Rights”.* Additionally, it considers that the Constitution must *“guarantee direct participation of these groups in political and administrative processes that may affect them”.* This proposal was supported by the *Fundación Chile Migra* (‘Chile Migrates Foundation’) and was the most popular initiative concerning migrants. The initiative “Asylum against oppression” (1.6) (549 votes) also argues for the inclusion of asylum as a fundamental human right, emphasizing the principle of non-refoulement. It was supported by the *Servicio Jesuita a Migrantes* (‘Jesuit service to migrants’) and states that Chile should *“advance towards an intercultural, fair and dignified society for everyone”.*

From a more concrete point of view, the initiative “The State’s social duty with people in need of shelter” (1.7) (36 votes) raises awareness on the lack of shelters for people in need. It argues that *“the State and society have the duty to advance, strengthen and improve the quality of life of any person in need for shelter, be they foreign or national citizens”* and that this should be done by way of building more comprehensive, integrated, and safe refuge centers.

With regards to alignment with wider frameworks, the proposals on human rights mainly align with the following Health Goals 2030: 1. Environmental health and healthy environments, 4. Non-communicable chronic diseases and Violence and 7. Management, quality, and innovation. In relation to the WHO Global Action Plan, these proposals are aligned with Priority 3: ‘Advocate the mainstreaming of refugee and migrant health into global, regional and country agendas and the promotion of: refugee-sensitive and migrant-sensitive health policies and legal and social protection (…)’ and Priority 4: ‘Enhance capacity to tackle the social determinants of health and to accelerate progress towards achieving the Sustainable Development Goals’.

### Category 2: regularization and naturalization of migrants

This category includes proposals related to naturalization processes in Chile. Opposing initiatives are seen, where one supports that obtaining the Chilean nationality should be facilitated and more expedient, and the other suggests that stricter measures should be put in place. A third proposal raises the idea of creating a new administrative body to help assist in the regularization process of migrants, and a fourth one broadly alludes to the concept of nationality, referring to respecting the nation and identifying with the homeland.

“We should all be Chilean” (2.1) (37 votes), an initiative put forward by a Venezuelan resident in Chile, touches on the fact that recent laws have made it more difficult to obtain the Chilean nationality. It suggests that there should not be discrimination based on residential visas and that, ideally, *“nationality could be granted to those who can demonstrate two years of continuous residence on the national territory”.* This proposal emphasizes the importance of *“being recognized as an equal by the State and society”.*

Also, in support of facilitating the regularization process, is the “Creation of an administrative dispute court” (2.2) (446 votes). This proposal advocates for the creation of an independent, administrative body which has the responsibility for (or capability of) *“recognizing and judging claims filed against the arbitrary acts or provisions of the administrative authorities”,* that would ideally lead to a regular migration process.

On the other hand, the initiative “Chilean nationality” (2.3) (158 votes)**,** raised by a Chilean citizen, suggests that irregular immigration is partly explained by the fact that any child born in Chile obtains the nationality (*ius solis* principle), generating a *“perverse incentive that facilitates illegal* (sic) *immigration”.* It argues that this principle should be changed in the new Constitution to the *ius sanguinis* principle, where at least one of the parents must have the Chilean nationality for the children to obtain it.

The first two proposals are aligned with Health Goals 2030 4. Non communicable chronic diseases and Violence and 7. Management, quality, and innovation, well as Priority 3 of the WHO Global Action Plan described above. The third proposal does not align with any guiding principles.

### Category 3: political participation and cultural integration of migrants and refugees

This category includes opposing initiatives concerning migrants' participation in political decision making. One initiative supports increasing the political participation of migrants, as well as recognizing their contribution to the country’s development, whereas another recommends increasing the minimum amount of time after which a foreign citizen can engage in electoral processes. In terms of cultural integration and identification matters, two proposals favor an inclusive approach towards migrants and minorities.

“Immigrant political rights” (3.1) (72 votes) establishes that “political inclusion of migrants is necessary to contribute to the development of a democracy that is able to represent the diversity of people and collectives that build society” and that “increasing their effective electoral participation is important so that political parties and candidates may consider them as active subjects within the voting groups”.

On the other hand, “Right to vote for foreign citizens starting from 10 years of obtaining permanent residence” (3.2) (107 votes)—as its name states—suggests that the right to vote for foreign citizens should only be conceded after 10 years of obtaining permanent residence. It argues that this would ensure a *“completely informed vote, considering the internal reality of the country and avoiding external political interference (…) with no externalities or unfunded fears”.*

Whereas the first proposal aligns with Health Goal 7 and Priority 3 mentioned above, the second one does not show associations with either framework.

A third proposal in this category is “Proposal to incorporate and recognize other flags and emblems belonging to indigenous communities, afro descendant tribes and migrant people'' (3.3) (23 votes). It recognizes *“the rights of diverse communities in our country, including some migrant groups with a determined representation, to use their own flags and/or their own emblems.*” Although it primarily focuses of indigenous communities and afro descendant tribes, it states that this would be a way to acknowledge* “the contribution of other migrant communities to the development of our country”.*

In broader terms, “Our nationality” (3.4) (140 votes)**,** raises the notion that *“we are all part and members of a nation that should be recognized and respected by all. One flag and emblem that is respected as such”*, irrespectively of our background, and therefore have the same rights and obligations. It suggests that the new Constitution must state that “*any person who born in Chile or who has fulfilled the necessary requirements, is Chilean”*.

Following the trend of the previous proposals, the first in this segment aligns to Health Goal 7 and Priority 3, whereas the second does not align with either.

### Category 4: specific regulations on slavery and human trafficking

In this category, both proposals refer to human trafficking and slavery as contemporary forms of abuse that are often overlooked and where migrants and undocumented individuals are especially vulnerable. They suggest that a constitutional stand on these matters would allow for an exclusive allocation of human and economic resources necessary to address and overcome this pressing matter.

The proposal, “Prohibition of slavery” (4.2) (141 votes) states that ‘modern slavery’ is manifested through human trafficking, forced labor, and sexual exploitation. It mentions that these phenomena have increased due to the migratory crisis and restrictive migratory policies, whereby irregular migrants become *“people without value, not recognized as equal by the State nor their employers”.* It suggests that implementing the right not to be submitted to slavery in the Constitution would allow the legal and judicial system to *“prosecute these crimes and protect the victims, with a focus on human rights and gender”,* and demands “*the allocation of resources to implement laws and public policies on modern slavery”.*

From a local point of view, the proposal “Recognition of the existence of human trafficking associated with children and adolescent institutions” (4.1) (69 votes) criticizes the state’s lack of responsibility regarding investigation and legislation on human trafficking, specifically referring to cases reported within Chile’s SENAME (National Service for Minors). It mentions that current public policies make migrant population more vulnerable to suffer from this practice and establishes that *“the constitution should mandate the state to facilitate and dedicate exclusive funds to face human trafficking, particularly institution-related trafficking, with a focus on human rights”.*

Both these proposals align with Health Goals 1. Environmental health and healthy environments and 4. Non communicable chronic diseases and Violence, along with Global Action Plan priorities 1. “Promote the health of refugees and migrants through a mix of short-term and long-term public health interventions”, 2. “Promote continuity and quality of essential health care, while developing, reinforcing and implementing occupational health and safety measures”, as well as with priority 4 mentioned previously.

Overall, only one of the proposals reached the minimum number of votes to pass on to be discussed in the constitutional convention, and none of the proposals explicitly referred to migrant health. There are alignments with national and international frameworks, the more explicit ones regarding Health Goal 4. ‘Non communicable chronic diseases and Violence’ and Global Action Plan priorities 3. “Advocate the mainstreaming of refugee and migrant health into global, regional and country agendas and the promotion of refugee-sensitive and migrant-sensitive health policies and legal and social protection (…)” and 4. “Enhance capacity to tackle the social determinants of health and to accelerate progress towards achieving the Sustainable Development Goals, including universal health coverage”.

## Discussion

As recognized by the United Nations (UN), making or reforming a given Constitution is ‘an exceptional opportunity for a state to create a common vision of its future’ [22]. In that sense, considering that Chile’s present-day Constitution was created during a dictatorship, it seems understandable that people would want a new start. Regarding the topic of our study, the 1980 constitution makes scarce mention of migrants, refugees, or asylum seekers, aside from defining eligibility to nationalize and voting rights. It also does not directly allude to the specific protection of migrant human rights or particularities regarding healthcare [23]. Globally and historically, health policies and legal frameworks in the context of migrants have tended to be modeled on the ‘threats’ associated to this group (i.e., communicable diseases and reemerging diseases). However, a rights-based approach has been gaining ground, where migrants’ multidimensional vulnerabilities to adverse health outcomes and mortality are recognized, and cross-sector coordination becomes necessary for proper global health governance [24]. The ‘Los Angeles Declaration on Migration and Protection’ (2022) signed by state leaders at the IX Summit of the Americas revolved around "Building a Sustainable, Resilient, and Equitable Future" and focused on ‘creating conditions for a safe, orderly, human and regular migration, as well as strengthening the necessary frameworks for international protection and cooperation’ [25]. In this line, it is imperative to analyze migrant health through the lens of the social determinants of health, and to address these structural elements as public health issues.

In our first category of analysis, regarding the promotion and defense of the human rights of migrants and refugees, the majority were supported by pro-migrant institutions and votes were higher than other categories. This could reflect an increased awareness of the significant human rights challenges faced by this group, as well as effective advocacy by supporting movements. Globally, commitments have been made to protect migrants’ human rights as seen in the New York Declaration on Refugees and Migrants [26] and the Global Compact for Safe, Orderly and Regular Migration [27]. However, within each country, barriers to access to and exercise of these rights remain, as well as a lack of legal resources when these rights are violated [28]. In Chile, for example, until the present year (2022), immigration legislation depended on the law decree 1094 promulgated in 1975 and was orientated towards control and security of the national territory [29]. Although it underwent modifications throughout the years, only now was it replaced by a new law which incorporates the recognition of migrants’ protection of labor rights, access to health, social security and tax benefits, education, and own housing, among others [30]. Worlwide, a study which analyzed 193 different constitutions regarding migrants’ constitutional protection found that only 24% of them explicitly guarantee some aspect of general equality and non-discrimination to foreign citizens. The totality of these constitutions introduced said modifications in 1990 or later [31]. It therefore seems that steering towards the integration of migrant and refugee rights into constitutional law is an important step in guaranteeing equal rights of non-citizens in an era of unprecedented human mobility.

Another proposal in this same category referred to establishing equality as a right, by placing a ban on racism and discrimination. It is concerning, nonetheless, that it only reached a total of 55 votes. This could reveal that Chilean society falls behind other countries in terms of its acceptance of racial and ethnic diversity and inclusion. Our findings complement the results presented by the local *Bicentenario Survey (2021)*, which demonstrate an increased ‘perception of great conflict’ between Chileans and migrants, from the point of view of the local population, as well as a significant increase in the idea that immigration is excessive [32]. From migrants’ perceptions, another survey (*Migrant Voices Survey (2021))* showed a high prevalence of discrimination reported by foreigners, and was more pronounced among Afro descendants [33].

Racism and discrimination should be a public health concern, as they can have deep repercussions on both individual and structural levels in terms of determinants of health. Individually, racism and discrimination have long been associated with adverse effects on mental and physical health [34], and structurally they create barriers to access healthcare. One Chilean study reported mistreatment by health care workers (HCW) towards migrants in the form of infantilizing or blaming them for not following certain programs [35], while other local reports establish that racist or discriminatory behavior discourage migrants attendance to healthcare appointments [36], making it a necessary issue to address.

With regards to regularization issues, our findings show some support for guaranteeing a regular migration process and opposing opinions on the prerequisites for obtaining nationality. Countries often use nationality or legal status as a condition for who may and may not enjoy access to healthcare facilities [37]. However, in Chile, the right to healthcare and health insurance for international migrants is supported by recent legal dispositions set out by the Health Ministry [38]. This shows that, legally, progress has been made so that foreigners, regardless of their migratory status, may have the right to access healthcare and receive the associated benefits.

Despite these efforts, in Chile barriers associated with migratory status persist, and access to healthcare is lower among migrant populations when compared to locals [39–41]. This reality is more pronounced among those who have recently arrived and are in irregular situations or without health coverage. Statistically, the lack of health coverage reaches up to 15.8% amongst migrants, compared to only 2.2% among nationals [42], constituting a public health concern and reflecting that there are still improvements needed.

One way to effectively engage migrants into their host society and functioning systems, as stated by the International Organization for Migration (IOM), is to focus on having a strong integration policy, understanding integration as a two-way process which requires dialogue and mutual adaptation, as well as respect for a core set of values and tolerance [43]. Our findings show that proposals on integration focused on two main areas: political participation and acceptance of cultural diversity.

Proposals focusing on political participation were opposing. On one hand, a representative of the migrant community demonstrated the importance of promoting migrants’ political participation and, on the other hand, a Chilean citizen expressed their apprehension towards promoting foreigners' participation. In Chile, foreigners who have resided in the country for more than five years may exercise the right to vote, making it one of the five countries that grants voting rights to non-naturalized immigrants [44]. Despite this, only 20.9% of eligible migrant voters participated in the 2020 constitutional plebiscite, compared to 51.6% of nationals [45]. Additionally, only those who have been nationalized as Chileans for at least five years can register as candidates and run for political positions, as stated in article 14 of the current Constitution [23]. The relevance of political participation has been recognized by the Council of Europe as an element of successful integration, through which foreigners are encouraged to actively participate in decisions that affect them [46]. Specifically, regarding health, cross-sectional data from 14 European countries found that immigrants living in countries with high integration policy scores experience better health than those with poor integration policies and ‘exclusionist’ models [47]. In light of this, and considering our findings, efforts should be made towards promoting migrants' political participation which, not only would facilitate their integration, but would also improve their quality of life and perceived health.

Referring to cultural integration, proposals were also conflicting. While one of them supported acceptance of diversity, it seems problematic that it received the lowest number of votes among all the proposals included in our study. The contrary proposal held more than five times the number of votes, and picks up on a more nationalist tone, stating ‘we are all members of a nation and respect one flag and emblem’. This last ideology, as described by Goksel, could be explained by a ‘Western interpretation of citizenship based on national identity and commonality as the foundation of democracy’. According to his analysis, cultural pluralism and ethnic diversity have been increasingly perceived as a threat, due to differences sometimes considered ‘irreconcilable’ with standards of Western societies [48]. Nonetheless, there is a global understanding that the protection of cultural identity is crucial, being recognized as a universal right by the UN since 1966 [49]. Constitutionally, cultural life and identity have been incorporated as a right in countries such as Panama, Guatemala and New Zealand [50]. In Chile, this was given legal recognition via a decree promulgated in 2007, which has within its guiding principles ‘equal dignity and respect to all cultures’ [51]. Yet examples such as our findings demonstrate that some citizens are still unprepared to accept cultural diversity. In medical environments, the lack of intercultural skills among healthcare workers acts as a constraint for migrants to fully exercise their right to health [1]. This reality has been described in recent Chilean studies and identified as a priority within efforts towards guaranteeing healthcare for international migrants [52]. Awarding cultural identity a constitutional protection could favor the integration of the migrant population across different sectors, including health, by institutionally promoting respect and tolerance, together with avoiding assimilation.

Lastly, our findings show that there is recognition of modern slavery and human trafficking as a reality that particularly affects the migrant community. This is revealing because, although issues regarding Chile’s SENAME (National Service for Minors) have been present for years, human trafficking is scarcely documented in Chile, especially when compared to other countries of the region [53]. Modern slavery, which affects 40.3 million people worldwide, essentially refers to ‘situations of exploitation that a person cannot refuse or leave because of threats, violence, coercion, deception, and/or abuse of power’ and can largely be traced to migration [54]. It has been used as an umbrella term that covers forced labor and marriage, human trafficking, and other forms of extreme exploitation. Such situations lead to serious physical and mental health issues [55] and, due to its magnitude and prevalence, researchers have asserted that it is a global health concern which deserves more attention. On this topic, Zimmerman suggests a preventative approach in tackling the problem, ‘target(ing) the harm before it occurs’, and identifying underlying factors that exacerbate its occurrence, such as certain business models and weak labor governance [56]. Human trafficking and modern slavery in Chile are still uncommon, but given the current migratory crisis, are bound to surface. Creating public policies from a preventative point of view can play an instrumental role in protecting migrants and vulnerable individuals and, in this respect, having a constitutional framework to guarantee legal support is paramount.

Regarding broader local and international frameworks, most of the proposals align with the guiding principles of working towards constructing favorable environments for migrants to have healthier, safer, and dignified conditions, which suggests that civilians tend to be in favor with such commitments. There was no direct mention of health nor an explicit contemplation of social determinants of health, however, constructing favorable environments for migrants to have healthier, safer, and dignified conditions acts on the social determinants that shape the health of international migrants.

The final plebiscite in the matter of accepting or rejecting the Constitutional Convention’s proposal for a new Constitution took place on the 4^th^ of September 2022, and its results were striking. In a historical process, where the vote was obligatory, the rejection option won with 61,87% of votes (7.886.434 million people) vs 38,13% of approval (4.860.266 million people) [57]. Following this outcome, discussions on how to go forward with the constitutional process in Chile are ongoing, yet it seems clear that this is just a starting point. Considering this, we believe the results of this study can be a useful resource for future processes and discussions, where citizen’s opinions should be considered. One critical area is citizens’ views on the role and situation of migrants within Chilean society.

This study has several strengths and limitations. The Popular Initiative for Norms received a large volume of inputs, as it was a widespread, online platform. Although there was a preliminary filter to those proposals that did not follow certain criteria, there was no thematic organization of all the initiatives sent through. This study is the first of its kind to analyze citizen’s constitutional proposals related to social and health protection of migrants in Chile and link their associations to broader institutional frameworks.

However, there are various limitations to this study. Firstly, the platform only allowed for national citizens or foreigners in regular migratory status to put forth ideas. This excludes those in opposite, more vulnerable situations and therefore could fail to represent their point of view. Additionally, the platform does not give the option of uploading proposals in other languages, meaning that views from individuals that do not speak or write Spanish are excluded.

## Conclusions

Our findings show emerging awareness and acceptance of migrants in Chile, as it is a topic taken into consideration for the country’s new Constitution. However, despite the important rise in migratory fluxes during the last few years, it still does not seem to be a priority, nor does it resonate widely among Chile’s citizens. Equally, although we analyzed these proposals through the lens of the social determinants of health, this was not something that was put forward by those who formulated them.

With regards to alignment with wider national and international frameworks, the proposals predominantly align with the Chilean Ministry of Health Goals that prioritize providing healthy environments and preventing violence, and the WHO Global Action Plan priorities of generating refugee-sensitive and migrant-sensitive health policies and legal and social protection.

While there is an urgent and evident need to define strategies in addressing migrants’ health in Chile, this study demonstrates that we must start by the first step, which is acknowledging migrants as subjects of human rights and as a population group which requires tailored policies and initiatives. Although the new constitutional proposal in Chile was rejected in September 2022, this study suggests that there is some emerging civil awareness on the topic of migration that deserves further recognition and discussion.

## Supplementary Information

**Additional file 1.** 

## Data Availability

https://doi.org/10.6084/m9.figshare.21566052.v1.
